# The American Journal of Insanity; and, Report of the Pennsylvania Hospital for the Insane

**Published:** 1846-03

**Authors:** 


*The American Journal of Insanity.* Edited by the Officers of the New York State Lunatic Asylum, Utica: and,

*Report of the Pennsylvania Hospital for the Insane,* for the year 1845. By THOS. S. KIRKBRIDGE, M. D., Physician to the Institution.

This Journal, together with the Report, all go to show how much the condition of that most afflicted and distressing of all classes of disease, the poor lunatic, is improved—that all physical restraint and violence are nearly if not entirely abolished—and that the most kind, humane, persuasive, intellectual and moral treatment, is now being every where introduced and practiced, and with the happiest results.

